# A comprehensive study of circulating tumour cells at the moment of prostate cancer diagnosis: biological and clinical implications of EGFR, AR and SNPs

**DOI:** 10.18632/oncotarget.19718

**Published:** 2017-07-31

**Authors:** Ignacio Puche-Sanz, María J. Alvarez-Cubero, Manrique Pascual-Geler, Alba Rodríguez-Martínez, Miguel Delgado-Rodríguez, José L. García-Puche, José Expósito, Inmaculada Robles-Fernández, Carmen Entrala-Bernal, José A. Lorente, José M. Cózar-Olmo, María J. Serrano

**Affiliations:** ^1^ Bio-Health Research Institute (Instituto de Investigación Biosanitaria ibs.GRANADA), Complejo Hospitalario Universitario Granada (CHUG), Department of Urology, University of Granada, Granada, Spain; ^2^ GENYO, Centre for Genomics and Oncological Research, Pfizer, University of Granada, Andalusian Regional Government, Granada, Spain; ^3^ Division of Preventive Medicine and Public Health, CIBERESP, University of Jaén, Campus de las Lagunillas, Jaén, Spain; ^4^ Bio-Health Research Institute (Instituto de Investigación Biosanitaria ibs.GRANADA), Complejo Hospitalario Universitario Granada (CHUG), Department of Medical Oncology, University of Granada, Granada, Spain; ^5^ Lorgen G.P., S.L., Business Innovation Center - BIC/CEEL, Technological Area of Health Science, Granada, Spain; ^6^ Laboratory of Genetic Identification, Department of Legal Medicine, University of Granada, Granada, Spain

**Keywords:** prostate cancer, circulating tumor cells, androgen receptor, epidermal growth factor receptor, single nucleotide polymorphisms

## Abstract

Circulating tumor cells (CTCs) have been recently accepted as prognostic markers in metastatic prostate cancer (PCa). However, very few studies have analyzed their role in early-stage PCa. The aim of this research is to study the value of CTCs at the moment of PCa diagnosis and to identify different subpopulations of CTCs. Patients with PSA value > 4 ng/ml and clinical suspicion of PCa were included. Samples were collected immediately before prostatic biopsy. CTCs were isolated by immunomagnetic technique using a multi-CK specific antibody. Molecular expression of EGFR and AR in the tissue was analysed by real-time PCR. Up to eight different SNPs in patients’ blood DNA were studied.

In a total of 86 patients, the CTC detection rate was 18.6%. The sensitivity, specificity, positive and negative predictive values of CTCs to detect PCa was 14.2%, 78.4%, 31.2% and 57.4%, respectively. Up to 75% of CTC-positive patients were AR-negative. A direct association was found between the expression of AR in the prostatic tissue and the presence of CTCs in blood (p<0.05). We observed an inverse relation between the expression of EGFR in the tissue and the expression of AR in the CTCs. No significant association between SNPs and CTCs was found.

The low detection rate of CTCs in early-stage PCa limits their role as a diagnostic marker. Nevertheless, we show that they may hide important prognostic information. Overexpression of AR in the prostate may facilitate cell dissemination.

## INTRODUCTION

Prostate cancer (PCa) is the most frequent non-cutaneous cancer affecting men and the second leading cause of cancer-specific mortality in males in the Western world [1]. Prostate specific antigen (PSA) screening is the main method for the early diagnosis of PCa, but has a low specificity. As a result, prostate biopsy remains the gold standard test for definitive PCa diagnosis. Given the potential serious side effects associated with PCa treatment, along with concerns about overdiagnosis, there is an urgent need for the discovery of biomarkers to obtain better predictive and prognostic information at the moment of diagnosis [2]. For this purpose, an optimal knowledge of PCa molecular biology is needed.

In 2008, De Bono et al [3] reported, in a multicentre prospective study, that circulating tumour cells (CTCs) counting was an independent predictor of overall survival (OS) in patients with metastatic castration-resistant prostate cancer (mCRPC). Since then, numerous studies have demonstrated the prognostic and predictive ability of CTCs in metastatic PCa patients. Nevertheless, only a few have analysed their role in localised PCa patients, mainly due to the very low counting rates in non-metastatic stages [4-7]. Adequate characterisation or phenotyping of CTCs, based on current knowledge of PCa biology, may be the key to overcoming this problem [8].

PCa is known to express high levels of androgen receptor (AR), and multiple mechanisms are involved in the maintenance of AR signalling, including PSA or epidermal growth factor receptor (EGFR) [9]. Evidence from several groups indicates that EGFR contributes to enhanced AR activity in PCa [10] and [11]. Furthermore, several genetic germline polymorphisms have been associated with PCa aggressiveness and risk of biochemical recurrence [12]. Among these genes, RNASEL (Ribonuclease L), ELAC2 (ElaC Ribonuclease Z 2) and MSR1 (Macrophage Scavenger Receptor 1) are the main genes related to PCa progression and aggressiveness.

The objective of this work is to better understand the role of CTCs at the moment of PCa diagnosis. For that, we determined well-known PCa-related markers (EGFR, AR) and genetic germline polymorphisms (rs56250729, rs486907, rs627928, rs11545302, rs17552022, rs5030739, rs4792311 and rs3747531) and studied their relationships with the presence of CTCs in peripheral blood. Furthermore, we characterised these CTCs according to their AR expression status.

## RESULTS

We analysed blood and biopsy samples from a total of 86 patients who met inclusion criteria.

### CTC counting

Firstly, we assessed the presence of CTCs in this cohort of patients. Out of the 86 patients, 70 (81.4%) were CTC-negative and 16 (18.6%) were CTC-positive, the majority of them with just 1 or 2 CTCs (Table 1).

**Table 1:** Results of CTC counting in the 86 patients

Number of CTC (cells / 10mL)	Number of patients (%)
None	70 (81.4%)
1	6 (7.0%)
2	7 (8.1%)
3	1 (1.2%)
4	2 (2.3%)

The baseline clinical characteristics of the patients were stratified according to the presence or absence of CTCs (Table 2). Only a younger age was found to be significantly associated with the presence of CTCs (*p*=0.030). No significant association was found between CTCs status and the other clinical characteristics, including prostate volume, tPSA, fPSA, fPSA/tPSA or testosterone levels.

**Table 2:** Comparison of mean values for the different clinical characteristics of patients according to their CTC status

	CTCs negative Mean ± SEM (n=70)	CTCs positive Mean ± SEM (n=16)	p value
Age (years)	69.77 +/ 0.9	65.31 +/− 2.4	0.030
Prostate volume (mL)	48.42 +/− 21.21	56.58 +/− 40.53	0.871
tPSA (ng/mL)	11.74 +/− 9.64	10.66 +/− 6.77	0.336
fPSA (ng/mL)	1.05 +/− 0.56	0.98 + +/− 0.40	0.346
fPSA/tPSA ratio	0.16 +/− 0.97	0.14 +/− 1.21	0.235
Testosterone (ng/mL)	5.74 +/− 2.74	4.88 +/− 1.78	0.132

*CTCs negative*: patients with none CTCs detected. *CTCs positive*: patients with 1 or more CTCs detected. *tPSA*: total PSA. *fPSA*: free PSA. *SEM*: standard error of the mean.

### Detection of AR in the CTCs

Of the 16 patients in whom CTCs were detected, 12 (75%) had AR-negative CTCs and 4 (25%) had AR-positive CTCs. No significant associations were found between the expression of AR in CTCs and the characteristics of patients including age, prostate volume, PSA and testosterone levels. Similarly, no statistically significant association was found between the AR status of the CTCs and the presence/absence of cancer in the biopsies.

### CTCs as a diagnostic marker of prostate cancer

A total of 35 (40.7%) patients showed a positive biopsy for prostate cancer, whereas 51 (59.3%) were negative for cancer. Among those with a positive biopsy 5/35 (14.3%) were CTCs-positive, while 30/35 (85.7%) were CTCs-negative. Among patients with a negative biopsy, 11/51 (21.6%) were CTCs-positive and 40/51 (78.4%) were CTCs-negative (Table 3).

**Table 3:** CTCs and biopsy results

	Biopsy + (n=35)	Biopsy – (n=70)	
CTC +	5 (14.3%)	11 (21.6%)	16
CTC −	30 (85.7%)	40 (78.4%)	70
	35	51	86

According to this data, the sensitivity, specificity, positive and negative predictive values of CTC testing to detect PCa were 14.2%, 78.4%, 31.2% and 57.4%, respectively. *CTC +*: >1cell/10mL. *Biopsy +*: pathologic evidence of prostate cancer.

We did not find any significant association between CTCs and the most common pathologic findings, such as the number of cylinders affected, Gleason score, perineural invasion or D’Amico risk category (Table 4).

**Table 4:** Prostate cancer characteristics of biopsy + patients (n=35)

		CTC −	CTC +	p value
Number cylinders affected (mean)		8.06	5.8	0.157
Gleason score	6	15 (50%)	1 (20%)	0.227
	≥ 7	15 (50%)	4 (80%)	
Perineural invasión		4 (13.3%)	0 (0%)	0.523
Risk category	Low	12 (40%)	1 (20%)	0.482
	Intermediate	8 (26.6%)	3 (60%)	
	High	10 (33.4%)	1 (20%)	

### Expression of EGFR and AR in tissue vs CTC status

The molecular expression of EGFR and AR on the biopsied tissues was analysed in 55/86 patients (Supplementary Material).

The expression of EGFR in the tissue was neither associated with the presence/absence of cancer in the biopsies (p=0.950) nor with the CTCs status (p= 0.255). Interestingly, when characterising CTCs according to the presence of AR, we found that 100% of patients with AR-positive CTCs were EGFR-negative in the prostate cancer tissue, whereas up to 75% of patients with AR-negative CTCs presented EGFR-positive tissue (p= 0.5).

**Figure 1:**
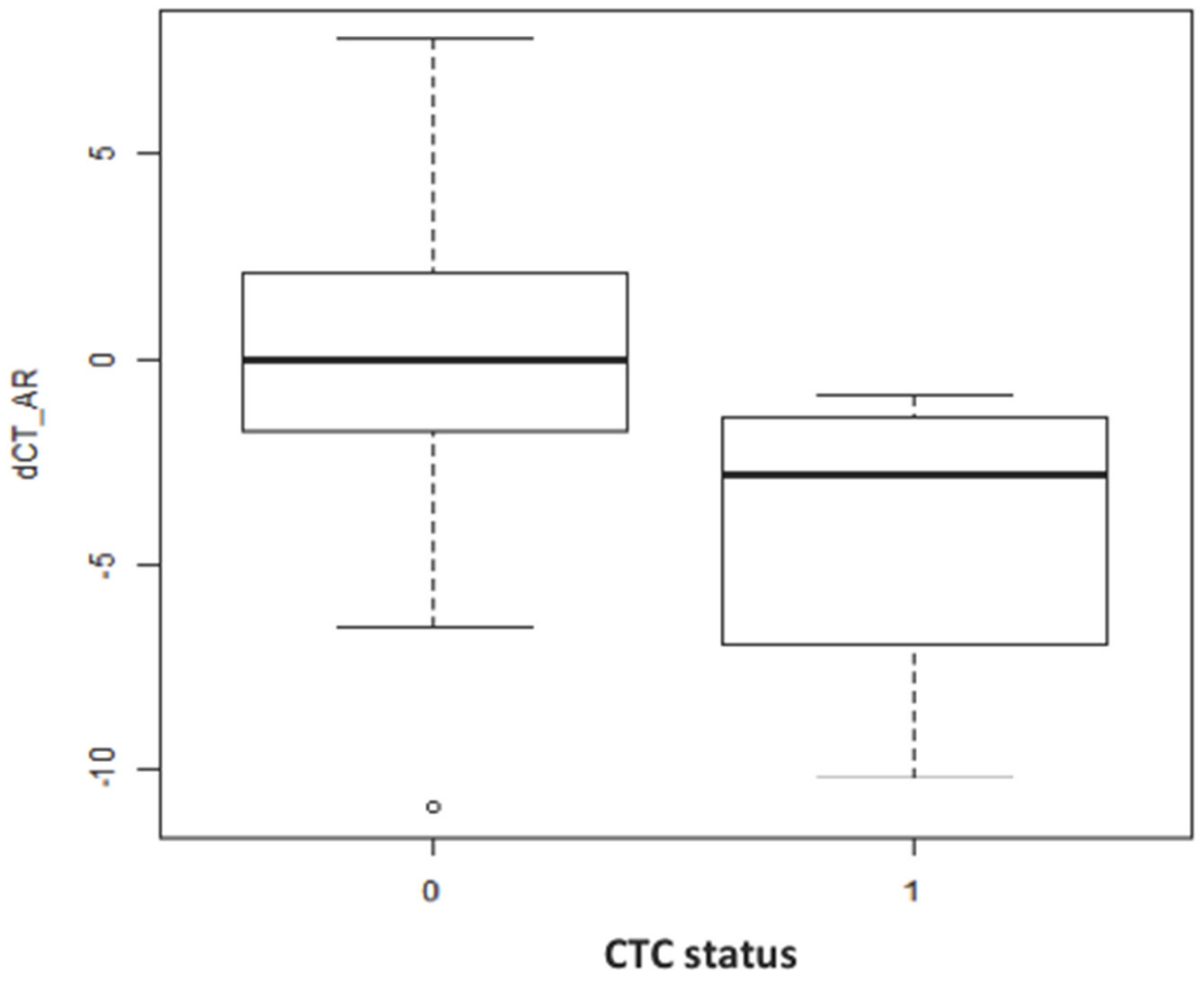
Box plot representation of AR expression in the prostatic tissue (dCT values) versus the CTC status (0: CTC-negative; 1: CTC-positive) (p=0.03) The line inside the plot represents the mean expression value of AR for each group. The higher the dCT values are, the lower the AR expression is.

Similarly, AR expression in tissue showed no association with the presence/absence of cancer in the biopsies (p=0.271). However, we found a positive relation between AR expression in tissue and the presence of CTCs (p=0.03) (Figure 1). When we compared AR expression in tissue with AR expression in CTCs, no significant association was detected (p= 0.347).

### SNPs vs prostatic biopsy and SNPs vs CTCs status

The genotype of SNPs was also analysed in 55/86 patients. Each single SNP was contrasted with the presence/absence of cancer in the biopsy and with the CTCs status. We did not find any statistically significant association (data not shown).

## DISCUSSION

To our knowledge, this work represents the largest analysis evaluating a set of biomarkers based on liquid biopsy from patients with elevated PSA and suspicious of PCa. Our study not only analyses the mere presence of CTCs, but also characterises them according to their AR expression. Furthermore, it assesses the relationship of CTCs with other markers in prostatic tissue as well as with several PCa SNPs.

CTCs have been largely studied in the metastatic setting, where their enumeration appears to be an important prognostic tool [3]. However, very few works have studied the prognostic role of CTCs in early-stage PCa. The studies on localised PCa have failed to demonstrate their clinical value [4-7]. One possible explanation of this lack of clinical impact could be the low number of CTCs that current technology allows to detect in the early setting, but it is also important to note that the number of studies in this population is insufficient and heterogeneous.

One of the most relevant studies in early-stage disease is that performed by Davis et al [5], who examined CTCs through the CellSearch® system in 96 patients with localised PCa prior to prostatectomy, and in a control group of 25 men with elevated PSA. They found CTCs in 21% of patients with localised PCa and in 20% of men with elevated PSA. The 18.6% detection rate in our series of patients with elevated PSA is similar to that obtained by this group. Likewise, they neither found any correlation between CTCs and tumour volume, pathological stage or Gleason score. Another recent study in 152 patients treated with radical prostatectomy also failed to demonstrate significant correlation between CTCs and PSA, Gleason score or the development of biochemical recurrence after 2 years follow-up [6].

Most studies demonstrate that CTCs do not correlate with the clinicopathologic characteristics of the primary tumour. So it was in our study, except for the age. Younger patients were more likely to have CTCs. This finding is consistent with other studies [14] reporting that patients younger than 60 had an increased number of CTCs, advocating a greater ease to disseminate due to an increased ability to proliferate in younger patients. These data suggest that it is the intrinsic tumour biology, and not the disease extent at diagnosis, what determines correlation with CTCs.

CTCs are a poor diagnostic marker of PCa [4] and [5]. Their number is low in early stages, and our results confirm this fact. However, the analysis of the mere presence of CTCs seems too simplistic, knowing their complex biology. CTCs characterisation is gaining importance to evaluate the ability of PCa to survive and progress [15]. For this reason, contrary to other studies that are only focused on their enumeration, we analysed the phenotypic characteristics of CTCs based on AR expression status.

Our results show that the majority of CTCs are AR-negative. Liu et al [15] suggested that the primary prostate tumour could harbour both AR-negative and AR-positive phenotypes. This finding is consistent with our data, as we detected CTCs with different AR expression patterns among our patients but also within one single patient sample. Thus, it is feasible that AR-positive primary tumours shed AR-negative CTCs and vice versa. This phenomenon has been detected in other hormone-dependent tumours, such as breast cancer [13], and has important implications for treatment strategies, as CTCs AR status could be a potential marker of response to AR-targeting therapies [17]. This AR heterogeneity in PCa may be an indicator of long-term progression to castration-resistant prostate cancer (CRPC) [18].

It is noteworthy the role of EGFR in the metastatic process and its relationship with the AR. AR can be indirectly activated by several growth factor receptors [9] and [10], mainly the EGFR and ERBB2. On the one hand, consistent evidence indicates that overexpression of EGFR contributes to PCa progression from hormone-dependent to CRPC [19-21], and that it is a promoter of the epithelial–mesenchymal transition (EMT) process [22]. On the other hand, an inverse association between AR expression in tissue and the acquisition of the EMT phenotype has been reported [23]. Finally, it is known that the EMT phenotype confers survival properties to cancer cells [24]. A recent *in-vitro* study in several PCa cell lines presenting different androgen sensitivities found that EGFR expression was highest in AR-negative cells [25]. Our results are consistent *in-vivo* with this statement, showing an inverse relation between EGFR expression in the prostatic tissue and AR expression in the CTCs. In other words, in our study most CTCs were AR-negative coming from EGFR-positive tissue. Thus, we hypothesise that AR-negative CTCs and EGFR-positive tissue might represent potential prognostic biomarkers due to their demonstrated EMT transformation ability.

It is widely accepted that androgens are required for PCa development and progression. We found a statistically significant association between the expression of AR in the tissue and the presence of CTCs, suggesting that an increase of AR expression in the prostate could facilitate CTCs dissemination into peripheral blood. This clinical finding is supported by recent experimental data, which demonstrate how androgens can induce EMT pattern in PCa cells, an essential molecular step that allows significant changes for cell migration and potential invasion [23].

With regard to germline genetic variants, it is clear that they are associated with PCa risk, as they may influence gene expression in prostatic tissue; however, their real prognostic role is uncertain, and controversial results are reported [26]. Here, we made a deeper analysis, being the first to include the effect of germline mutations in relation to expression patterns and CTCs presence. Although we found no statistically significant associations, a deeper analysis with an increased number of samples may give some relevant results.

### Limitations

We fully acknowledge that our results must be interpreted with caution, as the sample size is limited. One core limitation of the study is the low specificity of prostate biopsy. Although we tried to control this issue by performing systematic 20-core biopsies, the number of false negatives could still be high. A further follow-up of these patients over time is already scheduled, and some of these patients with elevated PSA could be diagnosed with prostate cancer in the future.

## MATERIALS AND METHODS

### Study design and patients

This is a cross-sectional study. Between May and December 2014, patients with clinical suspicion of PCa, based on individual PSA screening, and meeting criteria for prostate biopsy (PSA>10 ng/ml or PSA between 4 and 10 ng/ml with a free/total PSA <0.2) were enrolled in this study. Patients with a previous history of any type of cancer were excluded.

Immediately before the biopsy, all patients had 20 ml of peripheral blood collected for CTCs and genetic analysis. After that, all of them had a systematic 20-core transrectal ultrasound-guided biopsy. The laboratory technicians were blinded for PSA and biopsy results. The Ethics Committee Board of the Hospital approved the study and all patients provided written informed consent.

### Sample processing

#### Circulating tumour cells enrichment and detection

A total of 10 ml of blood was collected from each patient into CellSave Preservatives blood collection tubes (Veridex, LLC, Johnson & Johnson Company) before the biopsy. The samples were processed according to the protocol previously established by our group, using the Carcinoma Cell Enrichment and Detection Kit, MACS technology (Miltenyi Biotec,Germany) [13]. To identify CTCs, the samples were stained with antibodies specific to cytokeratin (CK) (CTCs^CK+^). Samples with high background staining were discarded. Details on sample processing are available in the Supplementary Material. CTC status was considered CTC-positive if ≥1 CTCs^CK+^/10 ml blood was detected.

#### Enumeration and characterisation of CTCs by CK and AR expression

**Figure 2:**
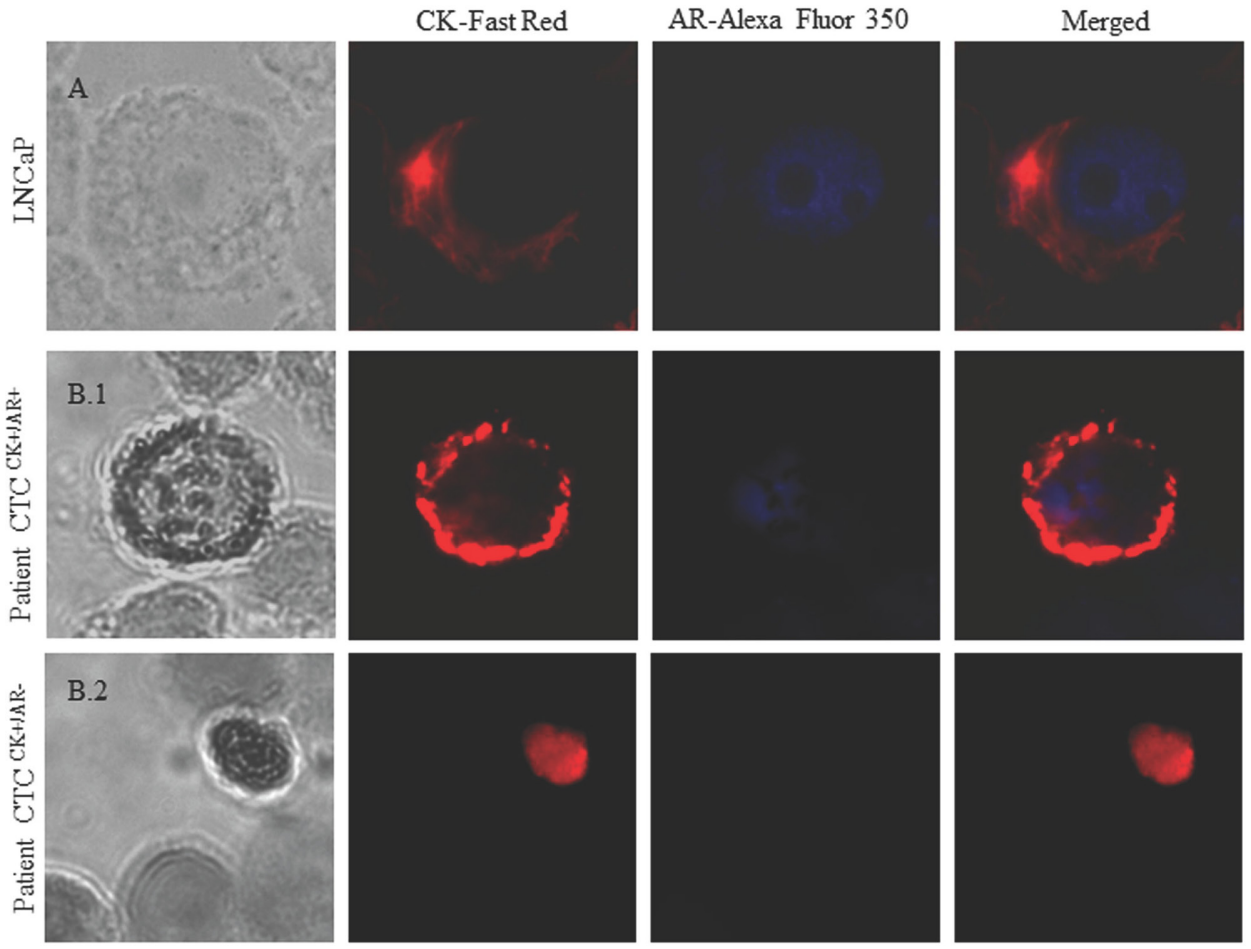
Image gallery after isolation, cytomorphological analysis and detection of cytokeratin-positive cells (CK+, red staining) and androgen receptor expression (AR, blue staining) **(A)** LNCaP cell tumour line was used as a positive control for AR expression. **(B)** Heterogeneous expression of AR in two different patients: **(B.1)** Patient 1 with positive AR expression in CTCs. **(B.2)** Patient 2 with negative AR expression in CTCs. AR-specific immunofluorescence (IF) CTCs was determined with Alexa Fluor® 350.

CK-positive and AR-positive cells were identified by immunohistochemistry (IHC), and the signal was detected by chromogenic and fluorescent detection, respectively, according to our protocol described in the Supplementary Material. Epithelial tumour cells were identified and enumerated based on their red staining for CK-positive cells and blue staining for AR-positive cells. Specific staining was easily distinguished because of the differential intracellular distribution of the examined molecules and the combination of direct and indirect immunofluorescence (IF) in order to evaluate CK+/AR+ expression in CTCs (Figure 2).

The performance of the assays was tested through the analysis of 17 healthy blood donors (HBDs) and prostate and cancer cell lines (LNCaP, PC3) (Supplementary Material).

#### mRNA expression of EGFR and AR in prostate tissue by real-time-polymerase chain reaction (RT-PCR)

Biopsied prostatic tissue was stored at -80°C until assayed. A pathologist selected a representative sample for this test. Tissue total RNA was extracted using Trizol procedures (Life Technologies, Foster City, CA, USA). Reverse transcriptase PCR of the RNA extracted was pre-amplified using RealTime ready cDNA Pre-Amp Master, in combination with RealTime ready Pre-Amp Primer Pools (Roche Applied Science, Indianapolis, IN). For details on the primers, see Supplementary Table 1.

EGFR and AR gene expression were assessed by quantitative RT-PCR on the 7900 Real-Time PCR System (Life Technologies, Foster City, CA, USA) based on SYBR® Green chemistry. Each test was run four times to avoid errors in expression analysis. To determine the relative expression levels of each gene, we applied the ΔCt method and normalised the data using two housekeeping genes: hypoxanthine phosphoribosyl transferase 1 (HPRT1) and glyceraldehyde-3-phosphate dehydrogenase (GADPH). Samples with Ct values >35 were excluded due to low quality or low amount of cDNA.

#### Single nucleotide polymorphisms (SNPs) genotyping

The genotype of 8 different SNPs (rs56250729, rs486907, rs627928, rs11545302, rs17552022, rs5030739, rs4792311, rs3747531) was analysed in patient’s blood DNA. These SNPs were chosen because they are included in the three main genes related to PCa aggressiveness and progression (RNASEL, ELAC2 and MSR1). Moreover, the selection of these 8 SNPs was done according to their prevalence in the Caucasian population.

For each reaction, 10 ng of genomic DNA were used along with 2 × TaqMan® Universal PCR Master Mix (Life Technologies). Details of the SNPs probe can be found in Supplementary Table 2. The PCR conditions were as follows: initial activation at 95 °C for 10 min, followed by 50 cycles of 15 s denaturation at 92 °C, and extension at 60 °C for 90 s. In total, 10% of the samples were amplified and sequenced by the Sanger method in a 3130 HIDI (Life Technologies, Foster City, CA, USA) to confirm data.

### Outcome measurements and statistical analysis

The presence of CTC was used as a diagnostic marker of the presence of PCa, and the results of biopsy were taken as the gold standard. In this situation, sensitivity, specificity and predictive values were estimated. In order to find different subpopulations of CTCs, we phenotyped them according to their AR expression.

We subsequently studied the relationships between the presence of CTCs and the main clinical and pathological variables, as well as between the presence of CTCs and the expression of PCa-related markers in prostatic tissue (EGFR and AR) and peripheral blood (SNPs).

For the statistical analysis, comparison of means was evaluated by the *t*-test. For categorical data, Fisher’s exact test was used. Real-time PCR data were expressed as ΔCt: the difference in Ct between the gene of interest and the mean Ct of two endogenous control genes, GADPH and HPRT1. The statistical significance in differential expression among groups was evaluated by the Wilcoxon test. All analyses were done in the R statistical environment.

## CONCLUSIONS

The low detection rate of CTCs in the early-stage setting limits their role as a diagnostic marker for PCa. Nevertheless, we demonstrate that they may hide important prognostic information showing that different subpopulations of CTCs can be found even in the first moments of the disease. Overexpression of AR in the prostate may facilitate cell dissemination. Further research with larger series of patients and a longer follow-up is warranted in order to understand the real role of CTCs at the moment that PCa is diagnosed.

## SUPPLEMENTARY MATERIALS TABLES



## Abbreviations

AR: androgen receptor
CK: cytokeratin
CPRC: castration-resistant prostate cancer
CTCs: circulating tumor cells
EGFR: Epidermal growth factor receptor
EMT: epithelial–mesenchymal transition
PCa: prostate cancer
PSA: prostate specific antigen
RT-PCR: real-time-polymerase chain reaction
SNPs: single nucleotide polymorphisms
