# Supplementary material for: A comprehensive study of circulating tumour cells at the moment of prostate cancer diagnosis: biological and clinical implications of EGFR, AR and SNPs
Source: Oncotarget. 2017 Jul 31;8(41):70472–80. doi: 10.18632/oncotarget.19718 (PMC5642570; doi:10.18632/oncotarget.19718)
Supplement: Supplementary file 1 [file oncotarget-08-70472-s001.pdf]

A comprehensive study of circulating tumour cells at the moment of prostate cancer diagnosis: biological and clinical implications of EGFR, AR and SNPs

SUPPLEMENTARY MATERIALS

Detection and characterization of CTCs isolated by immunomagnetic positive selection

Blood samples

After informed consent of patients, peripheral venous blood samples (10 ml) were collected in CellSave Preservatives Tubes (Veridex, LLC, Johnson & Johnson Company) and processed within 72 hr.

The study was reviewed and approved by the Hospital Ethics Committee. This way, blood samples from patients with high PSA levels and suspicion of PCa were extracted immediately before the prostate biopsy. As negative controls, 17 blood samples from healthy volunteers without evidence of an epithelial malignancy were examined. Positive controls were obtained using 10 ml of blood from healthy volunteers spiked with LNCaP, DU145 human prostate cancer line cells and processed separately from patient samples, to avoid contamination.

Density gradient separation

According to Sigma procedure, a gradient by layering 5 ml of HISTOPAQUE- 1119 (Sigma) in conical tubes was formed. Blood samples were carefully layered onto the HISTOPAQUE-1119 medium. Tubes were then centrifuged at 700*g* for 30 min. The GC and MNC fraction was found at the 1119 interphase. Then, cell fractions were mixed and washed in 50 ml PBS.

Magnetic labeling

Subsequently, cells were magnetically labeled according to the manufacturer protocols (Miltenyi Biotec, Bergisch Gladbach, Germany). Cytokeratin-expressing cells from peripheral blood of patients were enriched and detected using the Carcinoma Cell Enrichment and Detection Kit, Immunocytochemistry (Miltenyi Biotec). According to the manufacturer, cells were diluted in 35ml of 1X MACS Dilution buffer, then they were permeabilized by adding 5ml of MACS CellPerm Solution, incubating for 5 min at 20–25°C, and finally fixed upon addition of 5ml of MACS CellFix Solution with another incubation for 30 min at 20– 25°C. Cells were then washed twice in 1X MACS CellStain Solution and resuspended in 600 µl of the same solution. To block Fc receptors, 200 µl of FcR Blocking Reagent were added, and tumor cells were magnetically labeled by adding 200 µl of MACS Cytokeratin MicroBeads (colloidal super-paramagnetic MicroBeads conjugated to a monoclonal anti-cytokeratin 7/8 antibody; Clone: CAM5.2) and incubating for 45 min at 20–25°C. After incubation, cells were stained with 100 µl of anti-cytokeratin conjugated to fluorescein isothiocyanate (FITC) for 10 min in the dark at 20–25°C. After washing, cells were resuspended in a final volume of 500 µl CellStain Solution and stained with 10 µl of anti-FITC conjugated to alkaline phosphatase for 10 min in the dark at 20–25°C.

Magnetic cell separation

For magnetic enrichment of epithelial tumor cells, the magnetically labeled cell suspension was applied to a prefilled positive separation column (MiniMACS separation columns, type MS) in the magnetic field of a MiniMACS platform (Miltenyi Biotec).

Cells were passed through the column and washed three times with 500 µl 1X MACS Dilution Buffer, so negative (non-magnetic) cell population was washed out of the column. Afterward, the column was removed from the gradient magnetic field and the retained cells were collected (magnetic-positive cell population) as elute using the plunger after a dilution buffer washing step with a total volume of 1 ml.

Sample preparation and detection of epithelial tumor cells by immunocytochemistry

The magnetically enriched cell fractions were spun down onto polylysine-coated glass slides (Sigma) in a cytocentrifuge (Hettich, Tuttlingen, Germany) at 1,500 rpm for 10 min and the slides were air dried overnight at room temperature. Later, the slides were washed in 1X PBS and cytokeratin-expressing cells were revealed by incubation with freshly prepared Fast Red TR/Naphthol AS-MX substrate solution for 15 minutes in humidity chamber at room temperature. CTCs were identified by immunocytochemical methods and visualized under a direct light microscope to perform the combined cytomorphologic and immunophenotypic assessment. The cytomorphological criteria proposed by Meng et al. [1] (for example, high nuclear/cytoplasmic ratio, larger cells than white blood cells) were used to characterize a CK positive cell as a CTC.

Positive slides for CK-positive cells were washed in 1X PBS and stained with primary polyclonal goat

anti-human AR (Abcam) followed by incubation with polyclonal donkey anti-goat Alexa Fluor® 350 (Molecular Probes. Invitrogen). Epithelial tumour cells were identified and enumerated based on their red staining for CK-positive cells and blue staining for AR-positive cells. Specific staining can easily be distinguished because of the differential intracellular distribution of the examined molecules and the combination of direct and indirect IF in order to evaluate CK+/ AR. Identification and counting were done with a computerized fluorescence microscope Zeiss AXIO Imager.

Confocal microscopy

Confocal images were obtained using a Zeiss LSM 710 confocal/multi photon laser-scanning microscope equipped with Argon/2 laser (458, 477, 488, 514 nm) and a Titanium Sapphire laser (750 nm). The cells were viewed with several apochromatic objectives and images of different fields were taken. The images were captured using a spinning objective confocal microscope at X60 magnification.

Supplementary Table 1. All the primers were designed using Primer 3 software and synthesized by Sigma Aldrich®.

REFERENCES

1. Meng S, Tripathy D, Frenkel EP, Shete S, Naftalis EZ, Huth JF, Beitsch PD, Leitch M, Hoover S, Euhus D, Haley B, Morrison L, Fleming TP, et al. Circulating tumor cells in patients with breast cancer dormancy. Clin Cancer Res. 2004; 10:8152-8162. https://doi.org/10.1158/1078-0432.CCR-04-1110.

Supplementary Table 1: mRNA expression primers design

Name of the Gene	Sequence Primer Detail
**EGFR GENE (Epidermal growth factor receptor)**	
Forward	5′-TGCACCTACGGATGCACTG -3′,
Reverse	5′- CGATGGACGGGATCTTAGG -3′
**AR GENE (Androgen Receptor)**	
Forward	5′- GACATGCGTTTGGAGACTGC-3′
Reverse	5′- TTCCCTTCAGCGGCTCTTTT-3′
**GADPH GENE (Glyceraldehyde-3-Phosphate Dehydrogenase)**	
Forward	5′- ATCACCATCTTCCAGGAGCGAGA -3′
Reverse	5′- CATGGTTCACACCCATGACGAACA -3′
**HPRT1 GENE (Hypoxanthine Phosphoribosyl transferase 1)**	
Forward	5′- TGACACTGGCAAAACAATGCA-3′
Reverse	5′- GGTCCTTTTCACCAGCAAGCT-3′

* The molecular expression of EGFR and AR on the biopsied tissue was analyzed only in 55 out of the 86 patients due merely to a logistical issue.

Supplementary Table 2: Details of SNPs probes used for genotyping

Name of the Gene	Details of the probe
**RNASEL GENE**	rs56250729 (C__28997511_10)
	rs486907 (C____935391_1_)
	rs627928 (C___1914241_10)
**ELAC2 GENE**	rs11545302 (C__25981758_10)
	rs17552022 (C__25981570_10)
	rs5030739 (C__25981698_10)
	rs4792311 (C__25981579_20)
**MSR1 GENE**	rs3747531 (C__16172249_30)
